# Identification and functional prediction of cold-related long non-coding RNA (lncRNA) in grapevine

**DOI:** 10.1038/s41598-019-43269-5

**Published:** 2019-04-29

**Authors:** Pengfei Wang, Lingmin Dai, Jun Ai, Yongmei Wang, Fengshan Ren

**Affiliations:** 1 Shandong Academy of Grape, Shandong engineering research center for Grape cultivation and deep-processing, Jinan, 250100 P.R. China; 2 School of Pharmacy, Binzhou Medical University, Yantai, 264003 P.R. China; 3 Institute of special animal and plant sciences of CAAS, Changchun, P.R. China; 4 National Field Gene Bank for Amur Grapvine, Zuojia, P.R. China; 5 Key Laboratory of Urban Agriculture (East China), Ministry of Agriculture, Jinan, P.R. China

**Keywords:** Genomics, Abiotic

## Abstract

Plant long non-coding RNA (lncRNA) undergoes dynamic regulation and acts in developmental and stress regulation. In this study, we surveyed the expression dynamics of lncRNAs in grapevine (*Vitis vinifera L*.) under cold stress using high-throughput sequencing. Two-hundred and three known lncRNAs were significantly up-regulated and 144 known lncRNAs were significantly down-regulated in cold-treated grapevine. In addition, 2 088 novel lncRNA transcripts were identified in this study, with 284 novel lncRNAs significantly up-regulated and 182 novel lncRNAs significantly down-regulated in cold-treated grapevine. Two-hundred and forty-two differentially expressed grapevine lncRNAs were predicted to target 326 protein-coding genes in a cis-regulatory relationship. Many differentially expressed grapevine lncRNAs targeted stress response-related genes, such as CBF4 transcription factor genes, late embryogenesis abundant protein genes, peroxisome biogenesis protein genes, and WRKY transcription factor genes. Sixty-two differentially expressed grapevine lncRNAs were predicted to target 100 protein-coding genes in a trans-regulatory relationship. The expression of overall target genes in both cis and trans-regulatory relationships were positively related to the expression of lncRNAs in grapevines under cold stress. We identified 31 known lncRNAs as 34 grapevine micro RNA (miRNA) precursors and some miRNAs may be derived from multiple lncRNAs. We found 212 lncRNAs acting as targets of miRNAs in grapevines, involving 150 miRNAs; additionally, 120 grapevine genes were predicted as targets of grapevine miRNAs and lncRNAs. We found one gene cluster that was up-regulated and showed the same expression trend. In this cluster, many genes may be involved in abiotic stress response such as WRKY, Hsf, and NAC transcription factor genes.

## Introduction

In eukaryotes, many transcripts are non-coding RNAs (ncRNAs)^1,2^. Long ncRNA (lncRNA) is a type of ncRNA that is generally >200 nt long and has no discernable coding potential^3,4^. Most lncRNAs can be broadly classified into three types based on their genomic positions: (1) lncRNAs transcribed from intergenic regions of lncRNAs are known as lincRNAs (long intergenic non-coding RNA); (2) lncRNAs transcribed from introgenic regions are long intronic RNAs, which can be transcribed in any orientation relative to coding genes; and (3) long non-coding nature antisense transcripts (lncNAT) that overlap with protein-coding regions or ncRNAs on the opposite strand and antisense RNA^5–7^. In eukaryotes, different lncRNAs have been shown to be differentially expressed in different tissues or under different stress conditions. This indicates that lncRNAs undergo dynamic regulation and act in the regulation of development and stress response^8^. LncRNAs have been shown to be involved in gene silencing, the control of flowering time, photomorphogenesis in seedlings, organogenesis in roots, and reproduction in plants^4,9–16^. Some lncRNAs can also serve as precursors to small RNAs^17–22^. Some lncRNAs can regulate proteins or microRNAs (miRNA) by acting as decoys that mimic target DNA or RNA. For example, the Arabidopsis microRNA target mimics the IPS1 lncRNA and the decoy ASCO-lncRNA^14,23^. This illustrates the competing endogenous RNA (ceRNA) theory, which is well-supported and is now widely accepted^17,24^. The ceRNA theory states that mRNA, lncRNAs, pseudogenes, and other miRNA sponges share common miRNA binding sites because the amount of any given miRNA is limited^24^.

Currently, growing evidence supports the view that non-coding RNAs, including lncRNAs, play important roles in regulating responses to a variety of abiotic and biotic stressors^25–27^. A previous study has identified 318 lncRNAs responsive to cold and/or drought stress in cassava^28^. In cotton, some lncRNAs were shown to possibly be involved in regulating plant hormone pathways in response to drought stress^29^. Several stress-responsive lncRNAs have been functionally characterized in plant signaling pathways such as lncRNA npc48^30^, At4/IPS1^23,31^, and npc536^32^. In addition, miRNA, another non-coding RNA, was shown to be involved in various abiotic stress responses such as cold stress (chilling or freezing) in plants^33–36^. LncRNAs compete with other miRNA sponges, such as target gene mRNA, to play important roles in eukaryotes^30,37–40^. Therefore, lncRNA may play important roles in various abiotic stress responses via the ceRNA mechanism.

Cold stress is an important environmental factor that negatively affects grapevine productivity and quality. However, in grapevine, the function of lncRNA and the relationship between grapevine lncRNA and cold stress or cold stress tolerance are unknown. Here, cold-inducible lncRNAs in grapevine were detected using RNA-sequencing and analysis. The potential function of these lncRNAs, their target genes, and the relationship between grapevine mRNAs, lncRNAs, and miRNAs were also predicted and analyzed. Our aims were to identify the cold-responsive lncRNAs and determine if or how cold stress response in grapevine is related to lncRNA regulation.

## Results

### Data mining of transcriptome sequencing and identification of lncRNAs in grapevine

**Figure 1:**
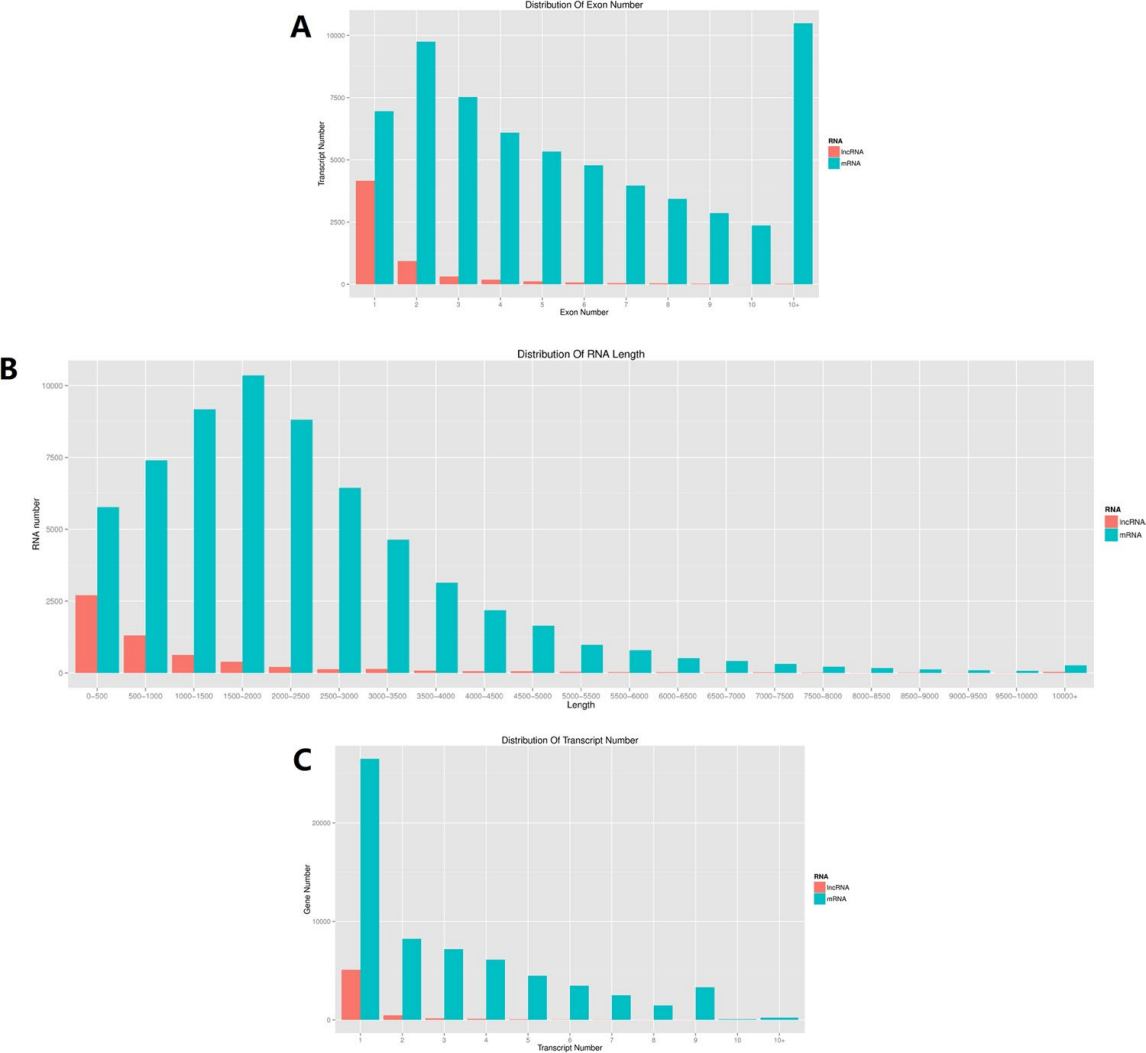
Characteristics of grape lncRNAs. (**A**) The number of exons per transcript for all mRNAs and lncRNAs. (**B**) Transcript size distributions for all mRNAs and lncRNAs. (**C**) Distribution of mRNAs and lncRNAs along each chromosome.

To systematically identify lncRNAs related to cold stress in grapevine, we performed whole transcriptome RNA-seq of grapevine cv. Cabernet Sauvignon that had been submitted to a cold-stress treatment of 4 °C. We generated an average of 12.65 gigabases (Gb) of raw reads per sample from the six samples used for Illumina RNA-sequencing. The total number of raw reads per control (CK) sample (plants were kept under a 16-h light/8-h dark photoperiod at 26 °C) ranged from 220842362 to 274931726, and the number of clean reads in each CK sample ranged from 216561108 to 270342092. The total number of raw reads in each cold treatment sample ranged from 191766324 to 233777742, and the number of clean reads in each cold treatment sample ranged from 186259776 to 223345340. The average mapping rate to the grapevine genome is 63.77%. In total, we identified 56732 transcripts, including 44644 known mRNA transcripts, 2031 known lncRNA transcripts, 7969 novel mRNA transcripts, and 2088 novel lncRNA transcripts. The transcripts of novel lncRNAs predicted here are listed in Table S1, and the transcript names and the related lncRNA gene IDs are listed in Table S2. The transcripts of novel mRNAs predicted here are listed in Table S3, and the transcript names and the related mRNA gene IDs are listed in Table S4. In all samples, we identified 212 novel lincRNAs, 1 933 novel long intronic RNAs, and 688 novel lncNAT. We also found 1 893 known lincRNAs, 511 known long intronic RNAs, and 803 known lncNAT in total samples. In addition, we found that it was the most common for the lncRNAs to contain only one exon; lncRNAs containing two exons were the next most common, followed by lncRNAs containing three exons and four exons (Fig. 1A). The lncRNAs less than 500 bp long were most common, followed by the 500–1000 bp long lncRNAs and 1000–1500 bp long lncRNAs (Fig. 1B). We also found that most lncRNAs were located on chromosome 1 (Fig. 1C).

### Variation in lncRNA expression among cold stress

**Figure 2:**
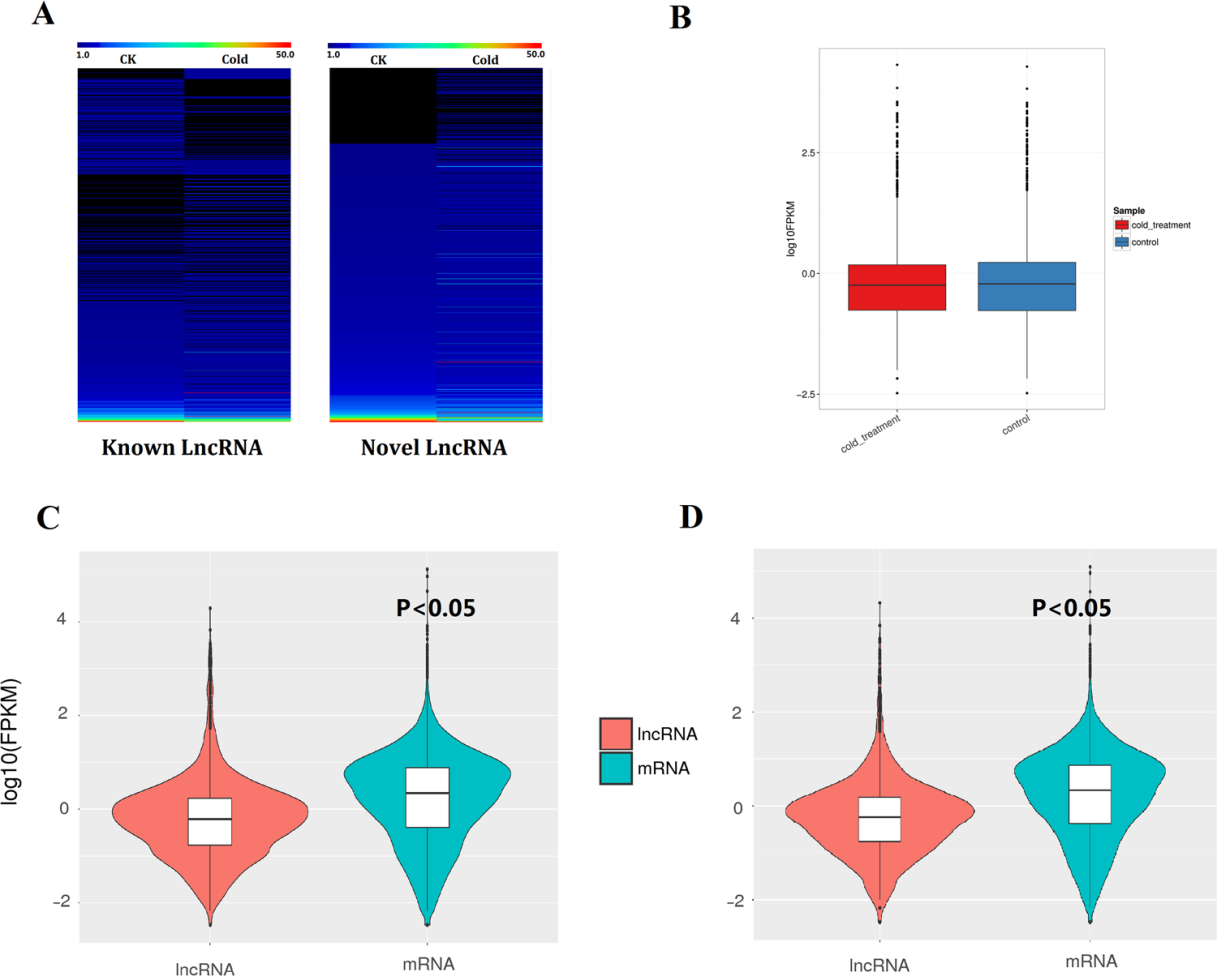
Expression models of grape lncRNAs and mRNAs. (**A**) The expression heatmap of all known and novel grape lncRNAs in the control and cold treatment based on the average FPKM value of each set of replicates. (**B**) The box plot of expression levels of grape lncRNAs under the control and cold treatment conditions. The y-axis represents the average log2 (FPKM) value of each set of replicates. (**C**) The violin map of expression levels of grape lncRNAs and mRNAs in the control. The y-axis represents the average log2 (FPKM) value of three replicates. *T-test* p-values < 0.05 are considered to be significantly different. (**D**) The violin map of expression levels of grape lncRNAs and mRNAs under cold treatment. The y-axis represents the average log2 (FPKM) value of three replicates. *T-test* p-values < 0.05 are considered to be significantly different.

In grapevine, 17 known lncRNAs were expressed only in the CK library and 97 known lncRNAs were expressed only in the cold-treated library. The expression heatmaps of all known and novel grape lncRNAs in the CK and cold treatment based on the Fragments Per Kilobase Million (FPKM) model are shown in Fig. 2A, and the box plot of expression levels of grape lncRNAs in the CK and cold treatment are shown in Fig. 2B. In both the control and cold treatments, the average expression level of the total lncRNAs was lower than that of the mRNAs in grapevine (Fig. 2C,D).

**Figure 3:**
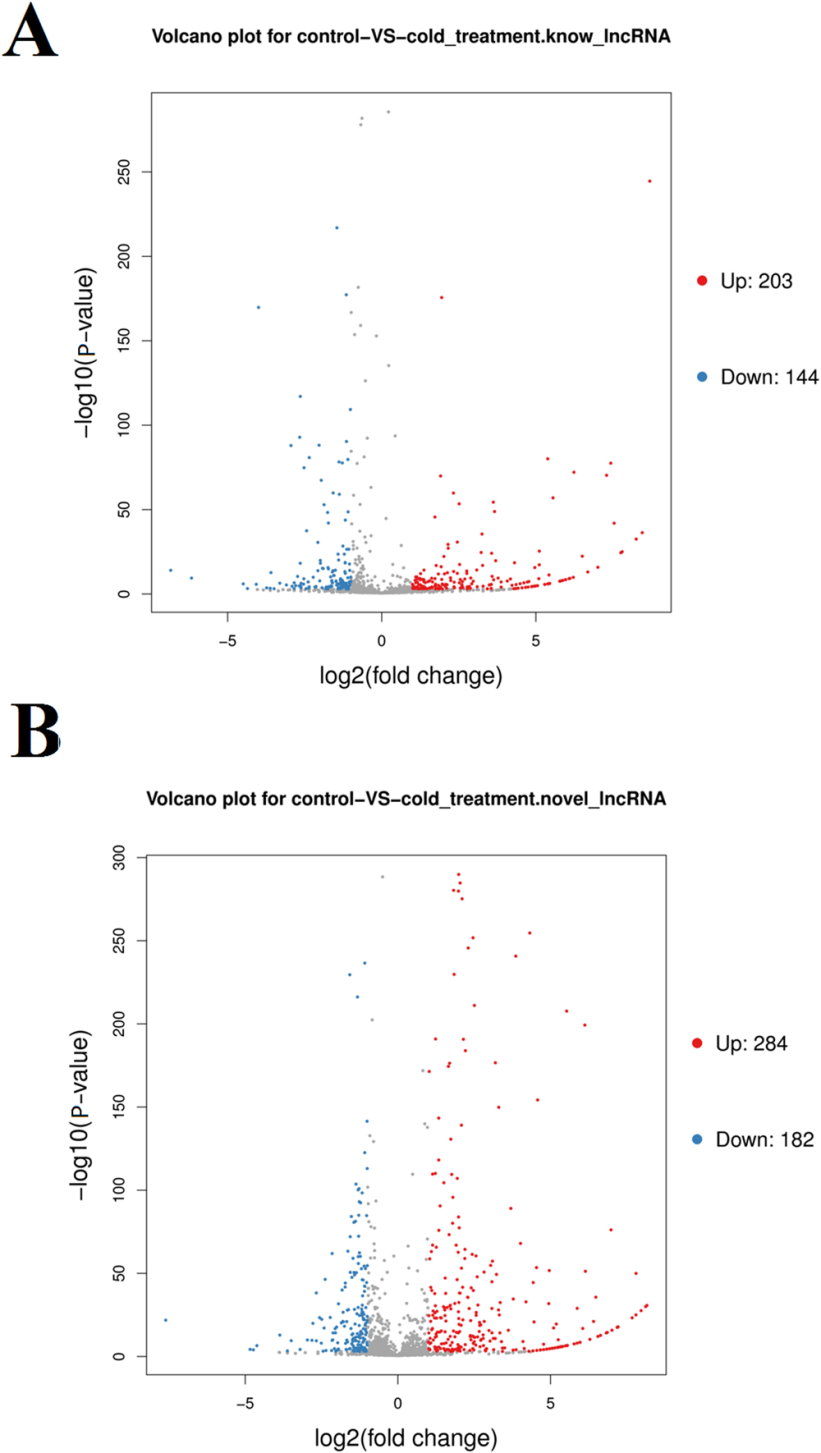
Differentially expressed lncRNAs in grapevine treated with cold stress. (**A**) The Volcano map of differentially expressed known lncRNAs and (**B**) differentially expressed novel lncRNAs. The x-axis represents the log2 (FPKM) values of the differentially expressed lncRNAs, and the y-axis represents the −log10 (P value) values of the differentially expressed lncRNAs.

Two-hundred and three known lncRNAs were significantly up-regulated (fold change > 2, P < 0.05) and 144 known lncRNAs were significantly down-regulated in cold-treated grapevine (fold change < −2, P < 0.05). In grapevine, VIT_203s0017n00360 was the lncRNA with the greatest increase of up-regulation by the cold treatment, followed by VIT_207s0031n00070 and VIT_201s0011n00530. VIT_209s0002n00340 was the lncRNA with the greatest down-regulation by cold treatment, followed by VIT_213s0158n00020 and VIT_213s0067n00110. These significantly up- and down-regulated lncRNAs were considered the differentially expressed known lncRNAs (Fig. 3A, Table S5).

In grapevine, 17 novel lncRNAs were expressed only in the untreated library and 11 novel lncRNAs were expressed only in the cold-treated library. We identified 284 novel lncRNAs as significantly up-regulated (fold change > 2, P < 0.05) and 182 novel lncRNAs were significantly down-regulated (fold change > 2, P < 0.05) in cold-treated grapevine compared with in the CK. In grapevine, LXLOC_001173 was the lncRNA with the greatest up-regulation in the cold treatment compared with the CK, followed by LXLOC_004676 and LXLOC_028762. Compared with the CK, LXLOC_003867 was the lncRNA with the greatest down-regulation in the cold treatment, followed by LXLOC_011153 and LXLOC_017876. These significantly up- and down-regulated lncRNAs were considered the differentially expressed novel lncRNAs (Fig. 3B, Table S5).

### Prediction of target genes of cold-related lncRNA targets in cis-regulatory relationships

To investigate the possible functions of grape lncRNAs, we predicted the potential targets of lncRNAs in cis-regulatory relationships. We searched for known protein-coding genes located within 10 kb downstream and upstream of all the identified grape lncRNAs. These genes were thought to be the targets of lncRNAs in cis-regulatory relationships if the Pearson and Spearman correlation coefficients between the expression levels of these genes were ≥0.6 or ≤−0.6, and P < 0.05^41^.

**Figure 4:**
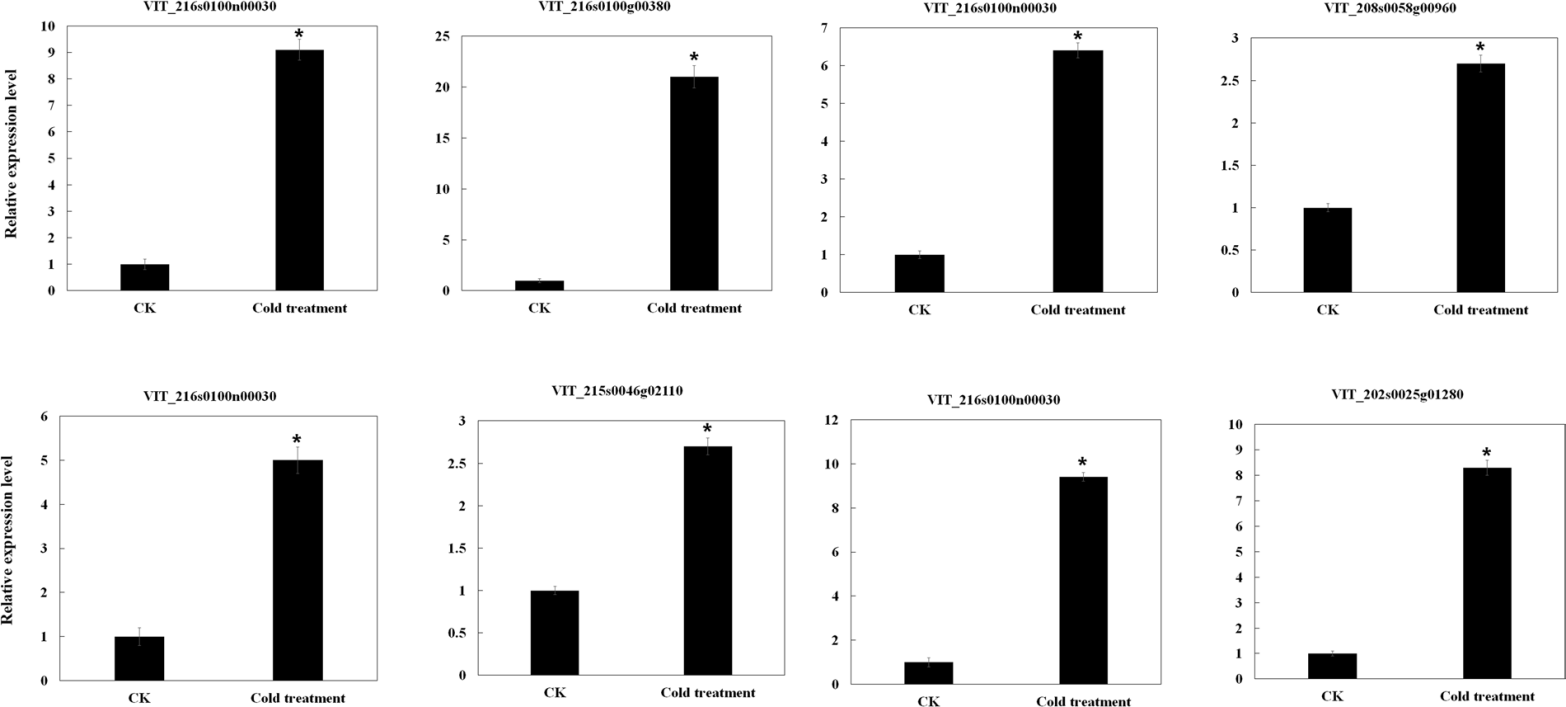
Expression level of cold inducible grapevine lncRNAs and their target genes validated by qRT-PCR. *T-test* p-values < 0.05 are considered to be significantly different, and “*” represents a p-value < 0.05.

**Figure 5:**
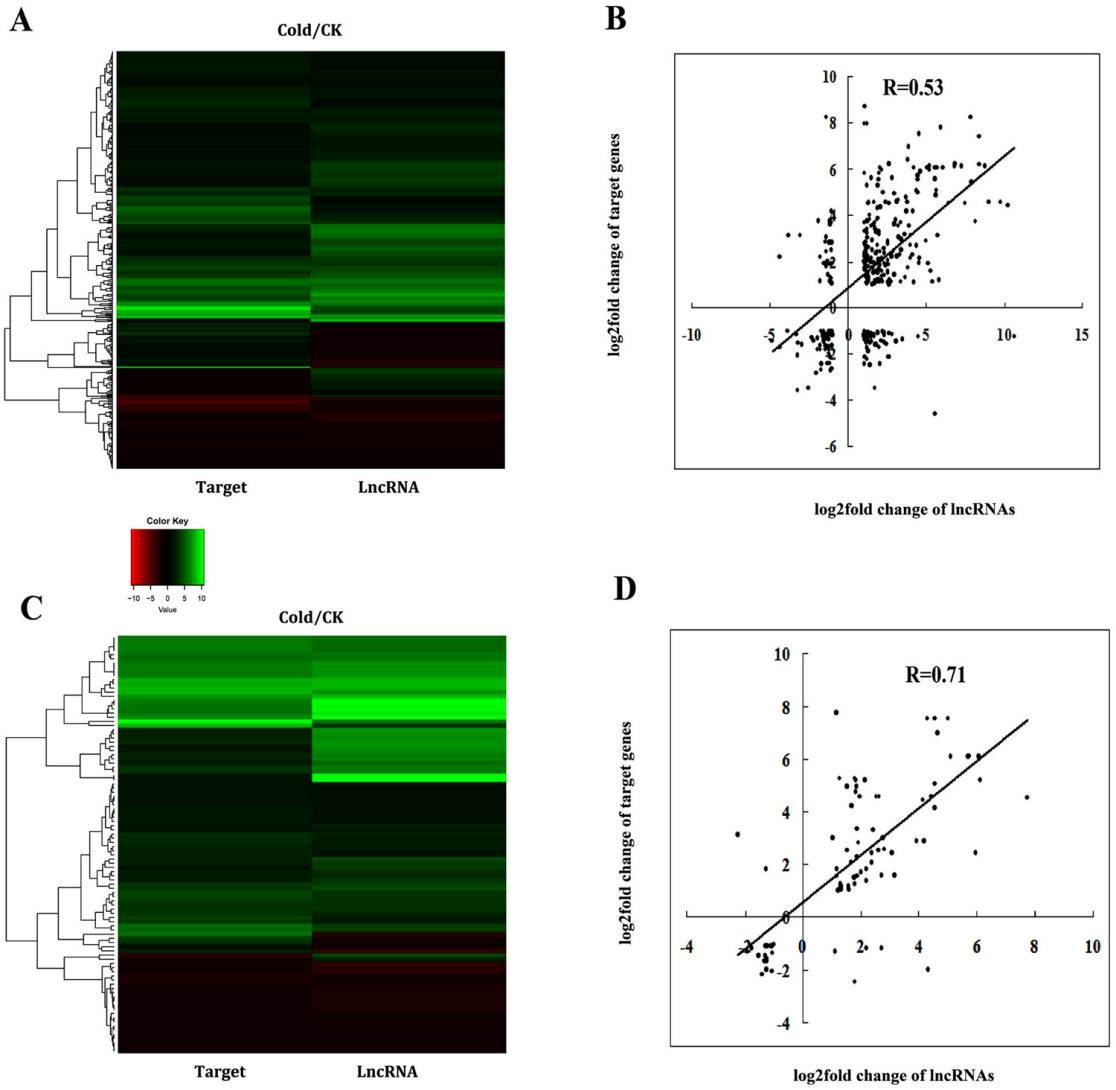
Comparison of the expression changes of differentially expressed lncRNAs, related target genes, and the correlation between them. The heatmap was generated from the fold change values in the RNA-seq data and was used to visualize the lncRNAs and cis-regulated relation target expression changes (**A**) and the lncRNAs and trans-regulated relation target expression changes (**C**). (**B**) The correlation between the expression changes of lncRNAs and cis-regulated relation target. The values along the x-axis are the log2fold change of lncRNAs (fold change = FPKM value of genes in the cold treatment/FPKM value of lncRNA genes in the control). The values along the y-axis are the log2fold change of their target genes in cis-regulatory relationships (fold change = FPKM value of genes in the cold treatment/FPKM value of genes in the control). (**D**) The correlation between the expression changes of lncRNAs and trans-regulated relation target. The values along the x-axis are the log2fold change of lncRNAs (fold change = FPKM value of genes in the cold treatment/FPKM value of lncRNA genes in the control). The values along the y-axis are the log2fold change of their target genes in trans-regulatory relationships (fold change = FPKM value of genes in the cold treatment/FPKM value of genes in the control).

Our results predicted a total of 2 527 target genes in cis-regulatory relationships of 1 650 lncRNAs in grapevine. In our study, significantly up-regulated or down-regulated lncRNAs were thought to be differentially expressed lncRNAs. Specifically, we found that 242 differentially expressed grapevine lncRNAs were predicted to target 326 protein-coding genes in cis-regulatory relationships, and many differentially expressed grapevine lncRNAs targeted stress response-related genes such as CBF4 transcription factor genes, late embryogenesis abundant protein genes, peroxisome biogenesis protein genes, and WRKY transcription factor genes (Table S6). The expression levels of some target genes in cis-regulatory relationships were positively related to lncRNAs. For example, VIT_216s0100n00030, LXLOC_027751, LXLOC_010422, and VIT_202s0025n00100 were up-regulated under cold stress compared to the CK. Based on our RNA-seq data, their target genes VIT_216s0100g00380 (CBF4 transcription factor), VIT_208s0058g00960 (transcription factor bHLH61), VIT_215s0046g02110 (late embryogenesis abundant protein Lea14-A), and VIT_202s0025g01280 (WRKY transcription factor 41) respectively, were also up-regulated under cold stress compared to the CK. The RNA-seq data was validated by the qRT-PCR results (Fig. 4). The expression levels of some target genes in cis-regulatory relationships were negatively related to lncRNAs. For example, compared to the CK, LXLOC_013001 was down-regulated under cold stress, but its target gene in the cis-regulatory relationship, VIT_217s0000g06350 (chlorophyll a-b binding protein 4), was up-regulated under cold stress when compared to the control. LXLOC_019156 was up-regulated under cold stress, but its target gene in the cis-regulatory relationship, VIT_202s0154g00610 (peroxisome biogenesis protein), was down-regulated under cold stress (Table S6, Fig. 5A). We calculated the correlation coefficient between the expression changes of lncRNAs and their target genes in cis-regulatory relationships under cold stress. As shown in Fig. 5B, the values of the x-axis are the log2fold change of lncRNAs (fold change = FPKM value of genes in the cold treatment/FPKM value of lncRNA genes in the control). The values along the y-axis are the log2fold change of their target genes in cis-regulatory relationships (fold change = FPKM value of genes in the cold treatment/FPKM value of genes in the control). The correlation coefficient was 0.53 (*t*-test, P < 0.05), indicating that the expression of overall target genes with a cis-regulatory relationship was positively related to the expression of related lncRNAs in grapevine under cold stress (Fig. 5B). The heatmap of expression of lncRNAs and their target genes in cis-regulatory relationships under cold stress based on the log2fold change value also showed that the expression of the overall target genes with cis-regulatory relationships were positively related to the expression of related lncRNAs in grapevine under cold stress (Fig. 5A).

### Analysis of target genes of cold-related lncRNAs in trans-regulatory relationships

To investigate the possible functions of grapevine lncRNAs, we predicted the potential targets of lncRNAs in trans-regulatory relationships. RNAplex software^42^ was used to identify the lncRNA (parameters: >−30 binding energy) as described in a previous study^41^. The Pearson and Spearman correlation coefficients between the expression of these genes identified using RNAplex and the expression of related lncRNAs must be ≥0.6 or ≤0.6 and P < 0.05, or will be filtered out^41^.

In grapevine, we predicted a total of 574 target genes in trans-regulatory relationships with 422 lncRNAs (Table S6). The results showed that 62 differentially expressed grapevine lncRNAs were predicted to target 100 protein-coding genes in trans-regulatory relationships such as NADH dehydrogenase subunit genes, UDP-glycosyltransferase genes, calcium-transporting ATPase genes, disease resistance protein genes, and glutamate receptor genes. However, most target genes in trans-regulatory relationships were unknown protein coding genes (Table S6). The expression levels of some target genes in trans-regulatory relationships were positively related with lncRNAs. For example, VIT_200s0225n00020 was down-regulated under cold stress, and its target gene, VIT_200s0246g00150 (NADH dehydrogenase subunit 5), in the trans-regulatory relationship was up-regulated under cold stress. Some target genes with trans-regulatory relationships were negatively related to lncRNAs (Fig. 5C).

We calculated the correlation coefficient between the expression changes of lncRNAs and their target genes in trans-regulatory relationships in the cold stress treatment. As shown in Fig. 5D, the values along the x-axis are the log2fold change of lncRNAs (fold change = FPKM value of genes in the cold treatment/FPKM value of lncRNA genes in the CK). The values along the y-axis are the log2fold change of their target genes in trans-regulatory relationships (fold change = FPKM value of genes in the cold treatment/FPKM value of genes in the control). The correlation coefficient was 0.71 (*t-*test, P < 0.05), indicating that the expression levels of overall target genes with trans-regulatory relationships were positively related to lncRNAs in grapevine under cold stress (Fig. 5D). The heatmap of expression of lncRNAs and their target genes in trans-regulatory relationships under cold stress based on the log2fold change value also showed that the expression of overall target genes with a trans-regulatory relationship were positively related to the expression of lncRNAs in grapevine under cold stress (Fig. 5C).

### GO enrichment and KEGG pathway analyses for differentially expressed lncRNA targets

**Figure 6:**
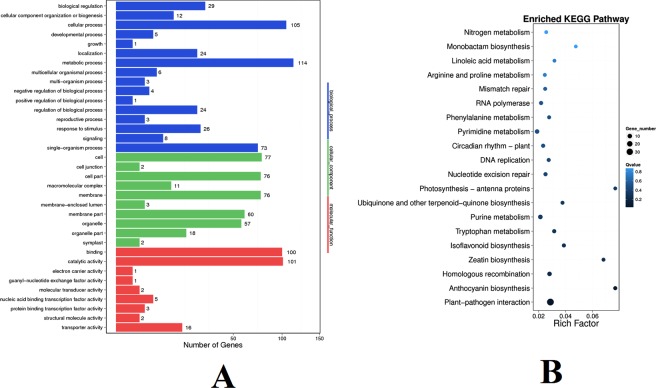
GO annotation and KEGG enrichment analysis of the differentially expressed target genes of lncRNAs. (**A**) The GO terms of the target genes of differentially expressed grapevine lncRNAs. (**B**) The top 20 enriched target genes of differentially expressed grapevine lncRNAs.

The potential function of grapevine lncRNAs in response to cold stress was studied using gene ontology (GO) annotation and enrichment analysis. Targets of differentially expressed cultivated grapevine lncRNAs were classified into three categories, 438 in biological processes, 231 in molecular functions, and 455 in cellular components. Biological processes contained 16 sub-categories with 299 terms, including the regulation of jasmonic acid mediated signaling pathway (GO: 2000022), regulation of defense response (GO: 0031347), regulation of signal transduction (GO: 0009966), hormone metabolic process (GO: 0042445), regulation of hormone levels (GO: 0010817), transmembrane transport (GO: 0055085), lipid metabolic process (GO: 0006629), chloroplast organization (GO: 0009658), flavonoid biosynthetic process (GO: 0009813), and flavonoid metabolic process (GO: 0009812). Molecular functions contained nine sub-categories with 156 terms, including chlorophyll binding (GO: 0016168), transcription factor activity, sequence-specific DNA binding (GO: 0003700), signal transducer activity (GO: 0004871), transcription factor activity, and transcription factor binding (GO: 0000989). Cellular components contained 10 sub-categories with 85 terms, including chloroplast (GO: 0009507), photosystem (GO: 0009521), chloroplast stroma (GO: 0009570), chloroplast envelope (GO: 0009941), and photosystem I (GO: 0009535) (Fig. 6A, Table S7). In molecular functions, the significantly enriched (P < 0.05) GO term was calcium ion transmembrane transporter activity (GO: 0015085). In target genes of differentially expressed grapevine lncRNAs, 87 KEGG (The Kyoto Encyclopedia of Gene and Genome) pathways were obtained and significantly enriched (P < 0.05) KEGG pathways included plant-pathogen interaction (ko04626), anthocyanin biosynthesis (ko00942), homologous recombination (ko03440), and zeatin biosynthesis (ko00908) (Table 1, Fig. 6B).

**Table 1:** Significantly enriched KEGG pathway of differential expressed grape lncRNAs.

KEGG Pathway	Pvalue	Pathway ID
Plant-pathogen interaction	1.9E-07	ko04626
Anthocyanin biosynthesis	0.00072	ko00942
Homologous recombination	0.00595	ko03440
Zeatin biosynthesis	0.01217	ko00908
Isoflavonoid biosynthesis	0.01363	ko00943
Tryptophan metabolism	0.01814	ko00380
Purine metabolism	0.02709	ko00230
Ubiquinone and other terpenoid-quinone biosynthesis	0.02882	ko00130
Photosynthesis - antenna proteins	0.03233	ko00196
Nucleotide excision repair	0.04746	ko03420
DNA replication	0.04943	ko03030

### Validation of lncRNA expression using qRT-PCR

**Figure 7:**
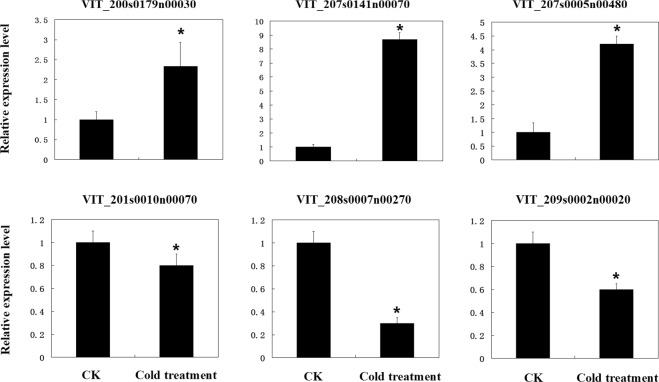
Expression level of select grapevine lncRNAs validated by qRT-PCR. *T-test* p-values < 0.05 are considered to be significantly different, and “*” represents a p-value < 0.05.

We performed qRT-PCR analyses to validate the RNA-seq results from six randomly selected grapevine lncRNAs, VIT_201s0010n00070, VIT_209s0002n00020, VIT_200s0179n00030, VIT_207s0141n00070, VIT_208s0007n00270, and VIT_207s0005n00480. The primers for qRT-PCR are listed in Table S8. The expression results were similar to the deep sequencing data (Fig. 7). VIT_200s0179n00030, VIT_207s0141n00070, and VIT_207s0005n00480 were shown to be up-regulated by the qRT-PCR data, showing a positive correlation with the deep sequencing results. VIT_201s0010n00070, VIT_208s0007n00270 and VIT_209s0002n00020 were down-regulated in both the qRT-PCR and RNA-seq results (Fig. 7, Table S5).

### LncRNAs as potential miRNA precursors

By aligning miRNA precursors to grapevine lncRNAs, we identified 31 known lncRNAs as 34 grapevine miRNA precursors, including vvi-MIR169h, vvi-MIR399a, vvi-MIR394b, vvi-MIR166a, and vvi-MIR156c (Table 2). We identified 25 novel lncRNA transcripts (19 lncRNA genes) as 22 grapevine miRNA precursors, including vvi-MIR162, vvi-MIR168, vvi-MIR535, vvi-MIR403a, vvi-MIR3623, and vvi-MIR3630. Some miRNAs may be derived from multiple lncRNAs. For example, vvi-MIR396b may be derived from VIT_211s0016n00330 and LXLOC_003224 (Table 2).

**Table 2:** grape miRNAs and the lncRNAs as their precursors.

MiRNA and lncRNA as precursor		MiRNA and lncRNA as precursor	
vvi-MIR156c	VIT_204s0008n00030	vvi-MIR162	LXLOC_012888
vvi-MIR159a	VIT_215s0046n00070	vvi-MIR162	LXLOC_012888
vvi-MIR159b	VIT_215s0046n00080	vvi-MIR164c	LXLOC_029337
vvi-MIR160c	VIT_210s0092n00020	vvi-MIR167a	LXLOC_000093
vvi-MIR164b	VIT_209s0002n00040	vvi-MIR167b	LXLOC_009023
vvi-MIR166a	VIT_208s0032n00030	vvi-MIR167b	LXLOC_007999
vvi-MIR166b	VIT_212s0034n00230	vvi-MIR167b	LXLOC_007999
vvi-MIR166c	VIT_215s0048n00320	vvi-MIR168	LXLOC_019119
vvi-MIR166d	VIT_216s0098n00100	vvi-MIR168	LXLOC_019119
vvi-MIR166e	VIT_202s0025n00230	vvi-MIR169g	LXLOC_028343
vvi-MIR166f	VIT_207s0031n00260	vvi-MIR169r	LXLOC_003511
vvi-MIR167d	VIT_200s0179n00030	vvi-MIR169t	LXLOC_003511
vvi-MIR167e	VIT_205s0020n00290	vvi-MIR169u	LXLOC_003511
vvi-MIR169y	VIT_201s0146n00060	vvi-MIR396b	LXLOC_003224
vvi-MIR169m	VIT_211s0103n00100	vvi-MIR396d	LXLOC_003867
vvi-MIR169r	VIT_211s0103n00110	vvi-MIR398a	LXLOC_000033
vvi-MIR169t	VIT_211s0103n00110	vvi-MIR535a	LXLOC_033356
vvi-MIR169u	VIT_211s0103n00110	vvi-MIR535a	LXLOC_033356
vvi-MIR171a	VIT_214s0068n00210	vvi-MIR535a	LXLOC_033356
vvi-MIR171b	VIT_212s0059n00020	vvi-MIR535b	LXLOC_033356
vvi-MIR394b	VIT_218s0001n00020	vvi-MIR535b	LXLOC_033356
vvi-MIR394b	VIT_218s0001n00020	vvi-MIR535b	LXLOC_033356
vvi-MIR396b	VIT_211s0016n00330	vvi-MIR535c	LXLOC_033356
vvi-MIR396d	VIT_211s0016n00340	vvi-MIR535c	LXLOC_033356
vvi-MIR399a	VIT_210s0003n00240	vvi-MIR535c	LXLOC_033356
vvi-MIR399b	VIT_216s0100n00020	vvi-MIR403a	LXLOC_022332
vvi-MIR169h	VIT_211s0103n00060	vvi-MIR403c	LXLOC_022332
vvi-MIR169i	VIT_211s0103n00070	vvi-MIR477a	LXLOC_033061
vvi-MIR169l	VIT_211s0103n00080	vvi-MIR477a	LXLOC_016789
vvi-MIR169n	VIT_211s0103n00100	vvi-MIR3623	LXLOC_014879
vvi-MIR169o	VIT_211s0103n00090	vvi-MIR3630	LXLOC_013003
vvi-MIR319e	VIT_211s0016n00290	vvi-MIR3630	LXLOC_013632
vvi-MIR394c	VIT_218s0001n00230	vvi-MIR3630	LXLOC_013003
vvi-MIR828a	VIT_216s0098n00140	vvi-MIR3633a	LXLOC_012920
vvi-MIR3636	VIT_216s0013n00110	vvi-MIR3633b	LXLOC_012920

### The relationships between grape mRNAs, lncRNAs, and miRNAs

We predicted the lncRNAs as targets or target mimics of miRNAs. In some previous studies, lncRNAs were found as both targets and target mimics of miRNAs^43,44^. As target mimics, lncRNAs could bind to miRNAs with a three-nucleotide bulge^43^. In our study, we only found lncRNAs that paired with miRNAs without any bulges. These lncRNA may be targets of miRNAs but not target mimics of miRNAs. Here, 212 lncRNAs as targets of miRNAs in grapevine were involved with 150 miRNAs (Table S9). Additionally, 120 predicted grapevine genes were both the target of grapevine miRNAs and lncRNAs (Table S10).

### Gene clusters show the same trends

**Figure 8:**
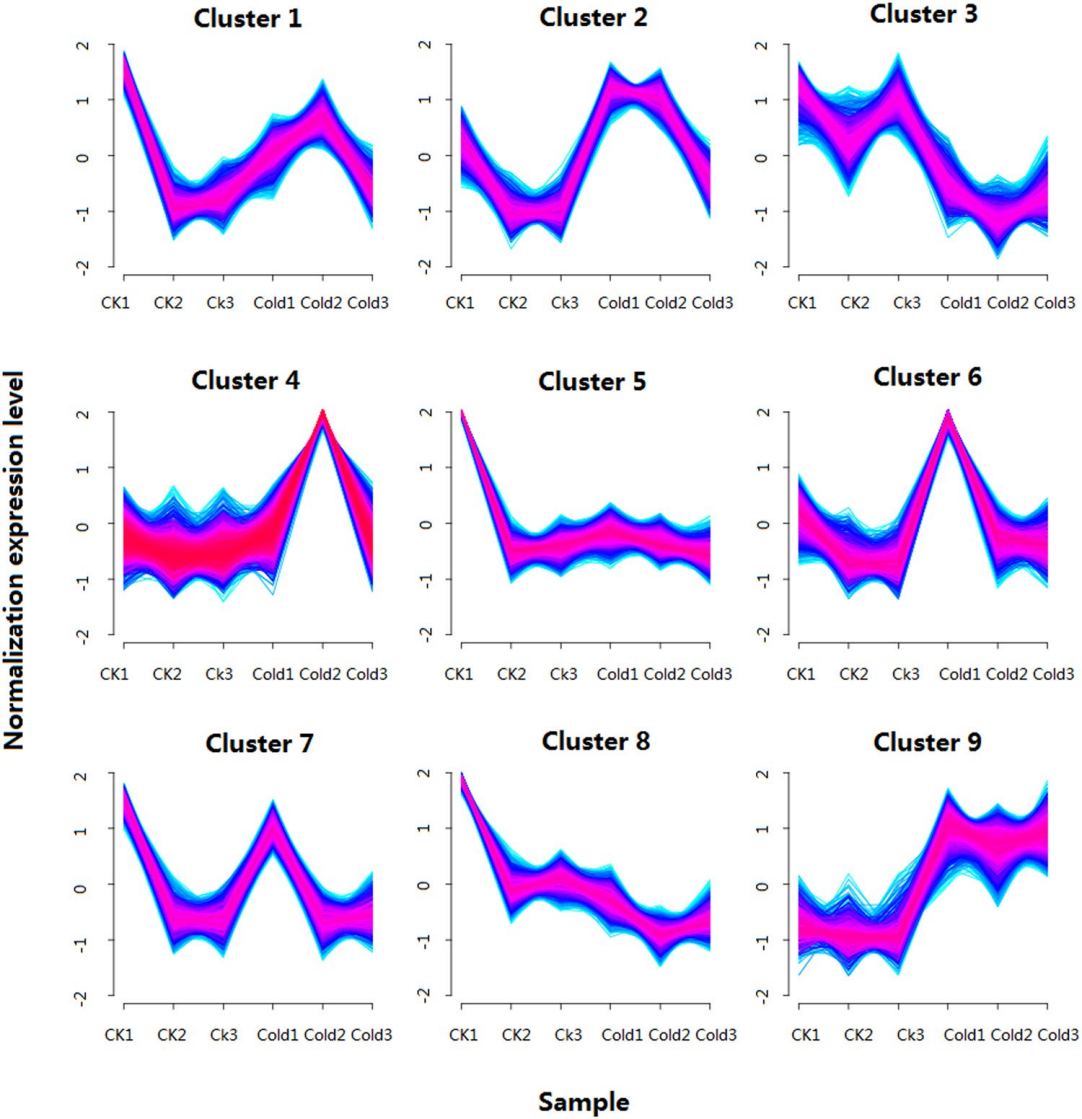
The expression pattern of genes from nine clusters in the control and cold treated samples.

**Figure 9:**
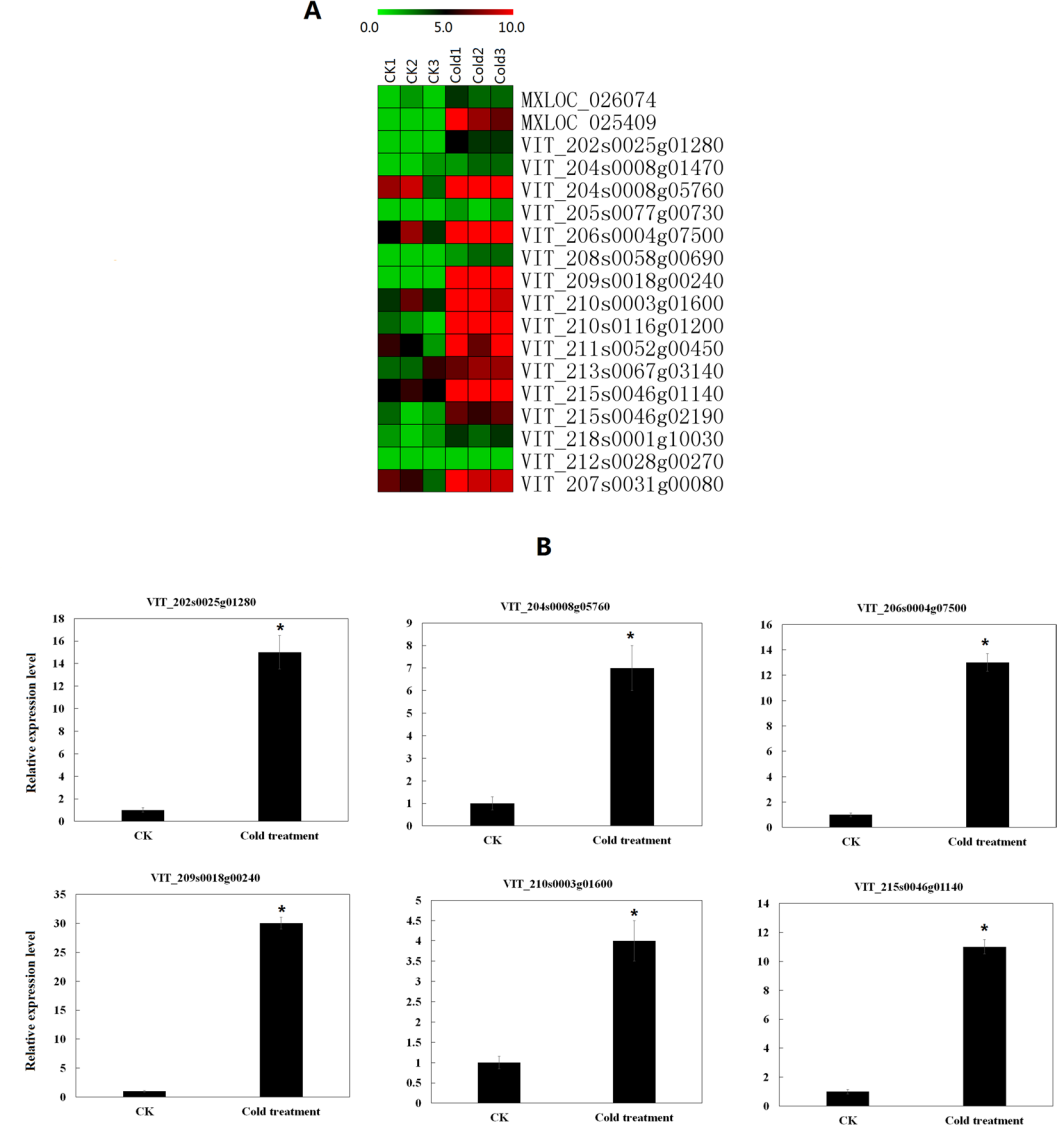
Expression level of WRKY genes in Cluster 9. (**A**) The heatmap was generated from the FPKM value of WRKY genes in Cluster 9 in each set of replicates. (**B**) Expression levels of some WRKY genes in Cluster 9 validated by qRT-PCR. *T-test* p-values < 0.05 are considered to be significantly changed, and “*” represents a p-value < 0.05.

The Mufzz software^45^ was used to cluster grapevine genes into gene clusters showing similar expression trends based on the expression changes of genes in cold treated grapevines. Nine clusters were identified and the genes with the same expression trend were clustered together (Fig. 8). Cluster 5 only contained 19 lncRNAs, Cluster 1 contained one lncRNAs, and Cluster 7 did not contain any lncRNAs. Clusters 2, 3, 4, 6, 8, and 9 contained more lncRNAs and their target genes than other clusters (Table S11). For example, Cluster 9 contained 137 lncRNAs and their 89 target genes. In cold treated grapevines, Cluster 9 showed an up-regulated expression pattern (Fig. 8), and in this cluster, 45 lncRNAs were significantly up-regulated under cold stress. In addition, 12 of their target genes were in Cluster 9, and the 12 target genes, which were significantly up-regulated under cold stress, contained LRR receptor-like serine/threonine-protein kinase, hydroxyacyl glutathione hydrolase, prolyl 4-hydroxylase subunit alpha-1, calcium-transporting ATPase 2, and some unnamed proteins (Table 3). Cluster 9 contained many ethylene-responsive transcription factor genes, such as two ERF5s and four ABSCISIC ACID-INSENSITIVE 5-like protein genes. Cluster 9 also contained NAM/ATAF/CUC (NAC) trans-transcription factor genes, such as NAC 68 and 94, as well as Hsf transcription factor genes, such as HsfA3, MYBA1, flavanone 7-O-glucoside 2′-O-beta-L-rhamnosyltransferase, isoflavone-7-O-methyltransferase 9, and WD repeat-containing protein (Table S11). Cluster 9 also contained 19 WRKY transcription factor genes including WRKY 3, 7, 11, 22, 28, 33, 40, 41, 46, 47, 48, and 50 (Table 4). RNA-seq data showed that 17 of these WRKY genes were significantly up-regulated (Fig. 9A), which was confirmed by the qRT-PCR (Fig. 9B). In cold-treated grapevines, Cluster 3 showed a down-regulated expression pattern (Fig. 8). Cluster 3 contained some ABSCISIC ACID-INSENSITIVE protein genes, auxin response factor genes, proline synthase co-transcribed bacterial homolog protein genes, NAC domain-containing protein genes, basic helix-loop-helix DNA-binding super family protein genes, cold-inducible RNA-binding protein genes, and WRKY transcription factor genes (Table S11).

**Table 3:** Up-regulated target genes belonged to cluster 9.

LnRNAs ID	Target genes ID	Gene annotation
LXLOC_000552	MXLOC_000552	solute carrier family 35 member B1
LXLOC_001158	MXLOC_000261	DUF246 domain-containing protein
LXLOC_001364	MXLOC_001364	unnamed protein product
LXLOC_001364	MXLOC_001363	unnamed protein product
LXLOC_001969	MXLOC_001969	LRR receptor-like serine/threonine-protein kinase
LXLOC_007796	MXLOC_008787	exopolyphosphatase
LXLOC_008601	MXLOC_008600	GPI ethanolamine phosphate transferase
LXLOC_011465	MXLOC_011465	unnamed protein product
LXLOC_019669	MXLOC_019669	unnamed protein product
LXLOC_024063	MXLOC_024063	hydroxyacylglutathione hydrolase 2
LXLOC_033142	MXLOC_033143	prolyl 4-hydroxylase subunit alpha-1
VIT_207s0129n00010	MXLOC_026393	calcium-transporting ATPase 2

**Table 4:** WRKY genes in cluster 9.

Genes ID	Gene annotation	Expressed change
MXLOC_026074	WRKY47	Up-regulated significantly
MXLOC_025409	WRKY33	Up-regulated significantly
VIT_202s0025g01280	WRKY41	Up-regulated significantly
VIT_204s0008g01470	WRKY50	Up-regulated significantly
VIT_204s0008g05760	WRKY3	Up-regulated significantly
VIT_205s0077g00730	WRKY48	Up-regulated significantly
VIT_206s0004g07500	WRKY33	Up-regulated significantly
VIT_207s0031g00080	WRKY7	No change
VIT_208s0058g00690	WRKY33	Up-regulated significantly
VIT_209s0018g00240	WRKY40	Up-regulated significantly
VIT_210s0003g01600	WRKY65	Up-regulated significantly
VIT_210s0116g01200	WRKY6	Up-regulated significantly
VIT_211s0052g00450	WRKY11	Up-regulated significantly
VIT_212s0028g00270	WRKY28	No change
VIT_213s0067g03140	WRKY70	Up-regulated significantly
VIT_215s0046g01140	WRKY46	Up-regulated significantly
VIT_215s0046g02190	WRKY22	Up-regulated significantly
VIT_218s0001g10030	WRKY7	Up-regulated significantly

## Discussion

A previous study reported the existence of lncRNAs in plants^37^. As next generation sequencing technology developed, it became possible to identify lncRNAs including those identified in Arabidopsis, rice, maize, cassava^4,8,28,46^, and grapevine (http://genomes.cribi.unipd.it/DATA/V2/V2.1/lncRNA/). However, few studies have been conducted on the roles of lncRNAs involved in abiotic and biotic stress responses. In addition, there has been limited research conducted on the roles of lncRNA involved in abiotic stress response, such as response to cold stress, in grapevine. In this study, we detected the expression changes of lncRNAs in grapevine exposed to cold treatment and found 2 088 novel grapevine lncRNAs. Previous studies have also identified novel lncRNAs in other plant taxa including 6 500 novel lncRNAs in *Arabidopsis thaliana*^8^, 1 704 novel lncRNAs in maize^45^, and 682 novel lncRNAs in cassava^28^.

Here, we found that the average expression level of the total lncRNAs was lower than the average expression level of mRNAs in grapevine in both the control and cold treatment conditions (Fig. 2C,D). This indicates that the expression levels of total lncRNAs should be lower than mRNAs in grapevine. In *A. thaliana*, approximately 300 lncRNAs were evidenced to be differentially expressed under abiotic stressors^27,31^, and 318 cassava lncRNAs were differentially expressed under cold and drought conditions^28^. Here, we found 813 differentially expressed grapevine lncRNAs in the cold stress treatment, showing that more grapevine lncRNAs were differentially expressed under cold stress. We hypothesize that many grapevine lncRNAs may be related to cold stress and may play important roles in cold stress response. Though the expression levels in many lncRNAs changed in the cold treatment, the average expression levels of the total lncRNAs in the cold treatment were similar to the average expression levels of the total lncRNAs under control conditions (Fig. 2B).

We predicted the target genes of cold inducible grape lncRNAs, finding more target genes of cold inducible grapevine lncRNAs in cis-regulatory relationships than in trans-regulatory relationships. This indicated that the target genes in cis-regulatory relationships may be more related to cold stress response. We also analyzed the expression correlation between the total cold inducible grapevine lncRNAs and their target genes, and our results showed that the expression patterns were positively related.

The expression correlation between cold inducible grapevine lncRNAs and their target genes in trans-regulatory relationships were higher than in cis-regulatory relationships. However, some of the expression patterns of lncRNAs were negatively related to their target genes (Fig. 3). A previous study showed that lncRNAs could act as enhancers of gene expression^47^. In kiwifruit, the expression of both protein-coding genes and lncRNA genes tended to be more positively correlated than negatively correlated in trans-regulatory relationships^48^. Here, we found that the expression of the overall target genes with a cis-regulatory relationship was also positively related to the expression of related lncRNAs in grapevine under cold stress.

Some target genes of cold inducible grapevine lncRNAs in cis-regulatory relationships may be involved in abiotic stress response such as VIT_216s0100g00380 (CBF4 transcription factor), VIT_215s0046g02110 (late embryogenesis abundant protein Lea14-A), and VIT_202s0025g01280 (WRKY transcription factor 41). These genes were also up-regulated in the cold stress treatment. Previous research has shown that CBF family genes play critical roles related to control of an important pathway in the cold acclimation process^49,50^. CBF4 is one of the most important members for the over-wintering of grapevines^50^. Some LEA proteins have been shown to be involved in the freezing tolerance of plants^51^. Additionally, some WRKY transcription factors have been shown to be involved in modulating gene expression in plants during cold stress^52^. These cold stress-related genes were also up-regulated under cold stress. Therefore, we hypothesize that these cold stress-related genes could be regulated by related lncRNAs under cold stress. These lncRNAs may play important roles in cold stress tolerance and may be related to the regulation of these cold stress-related genes.

The GO analysis showed that the biological process terms that are related to cold stress lncRNAs contained the regulation of jasmonic acid (JA) mediated signaling pathway (GO: 2000022), regulation of defense response (GO: 0031347), regulation of signal transduction (GO: 0009966), hormone metabolic process (GO: 0042445), and regulation of hormone levels (Fig. 6A, Table S7). Jasmonic acid is related to cold stress response in plants^53^. Other hormones, such as abscisic acid (ABA), are related to abiotic responses in plants^54^. Molecular function terms of genes that are related to cold stress lncRNAs contained transcription factor activity, sequence-specific DNA binding (GO: 0003700), and transcription factor binding (GO: 0000989) (Fig. 6A, Table S7), indicating that many target genes were transcription factors or were related to transcription factors. These transcription factors may be involved in cold response and the regulation of other downstream genes involved in cold response. Cellular component terms of genes that were related to the cold-related lncRNAs contained photosystem (GO: 0009521) and photosystem I (GO: 0009535) (Fig. 6A, Table S7), showing that many target genes may be related to photosystems. Under cold stress, the photosystems have been shown to be related to cold tolerance^55^.

We identified 31 known lncRNAs as 34 grapevine miRNA precursors, including vvi-MIR169h, vvi-MIR399a, vvi-MIR394b, vvi-MIR166a, and vvi-MIR156c (Table 2). In cassava, 12 lncRNAs were identified as 11 known cassava miRNA precursors, including miR156g, miR160d, miR166h, miR167g, and miR169d^28^. The lncRNAs that are precursors of miR156 and miR169 family members were identified in both grape and cassava^28^, indicating that some lncRNAs from different species might have been derived from same ancestral genes.

A previous study has shown that the lncRNAs that acted as target mimics could bind to miRNAs with three-nucleotide bulges^43^. However, our data did not predict similar target mimics that have been found in previous studies^43^, but the data did predict some targets that could bind to miRNAs without three-nucleotide bulges. These lncRNA may be targets of miRNAs. Similarly, a previous report has shown that lncRNAs acting as target genes could bind to miRNAs without bulges^44^. We found that lncRNAs and protein coding genes shared common miRNAs, which could target both lncRNAs and protein coding genes, and miRNAs and lncRNAs shared common target genes in grapevine. We hypothesize that the lncRNAs may regulate protein coding genes via complex pathways in grapevines.

The genes with the same expression trends were clustered together, and the genes in the same cluster may be involved in the same biological process^45^. We identified one cluster (Cluster 9) that showed an up-regulated expression pattern under cold treatment (Fig. 8). In this cluster, many genes may be involved in abiotic stress response such as WRKY transcription factor genes^51^, Hsf transcription factor genes^16^, and NAC transcription factor genes^56^. In Cluster 9, we also found 19 WRKY transcription factor genes, most of which were significantly up-regulated. Cluster 9 contained many lncRNAs and many protein coding genes that are the target genes of the lncRNAs in this cluster. Therefore, we suggest that the cluster may contain one or more pathways related to cold stress response and that lncRNAs may play important roles in cold stress response in this pathway. Because many WRKYs were found in Cluster 9, WRKY family members may play important roles in the key cold stress response pathway. Although none of the WRKY genes in Cluster 9 was a target gene of the lncRNAs, they may still be indirectly regulated by lncRNAs or regulated by the expression of lncRNAs; however, this requires further study. Additionally, in this cluster, there are some genes related to anthocyanin or flavonoid biosynthesis such as VvMYBA1, flavanone 7-O-glucoside 2′-O-beta-L-rhamnosyltransferase, isoflavone-7-O-methyltransferase 9, and WD repeat-containing protein (Table 11)^57–59^. Previous studies have shown that abiotic stressors (such as cold or heat stress) may regulate anthocyanin or flavonoid biosynthesis-related genes^57,58^. Anthocyanins have been shown to be synthesized as protective compounds in response to cold stress^60^. Our cluster analysis showed that some key anthocyanin biosynthesis related genes may be located in pathways involved in cold stress response; therefore, lncRNAs in pathways involved in cold stress response are related to these anthocyanin biosynthesis related genes. Supporting our findings, previous studies have shown that biotic or abiotic stressors are related to the biosynthesis of anthocyanins or flavonols in grapevine^58,59,61^. Further studies should be conducted on the relationship between anthocyanin/flavonoid biosynthesis pathways and cold stress as additional results will positively impact viticulture and breeding.

## Materials and Methods

### Plant materials

One-year-old self-rooted seedlings of the grapevine cv. Cabernet Sauvignon were grown and maintained in the greenhouse under a 16 h light/8 h dark photoperiod at 26 °C. For the cold stress treatment, plant materials under a 16-h light/8-h dark photoperiod were transferred to 4 °C for 4 hours. For the control (CK), plants were kept under a 16-h light/8-h dark photoperiod at 26 °C for 4 hours. The shoot apices with well-developed leaves from these plant materials were collected. Each treatment consisted of three independent replicates. RNA was isolated for the construction of RNA-seq libraries and real-time PCR analysis.

### Transcriptome library construction and high-throughput sequencing

Extracted RNA was sent to BGI (Shenzhen, China) for transcriptome library construction. In this process, RNA was treated with a Ribo-Zero™ Magnetic Kit to degrade rRNA. First-strand cDNA is generated by First Strand Master Mix and Super Script II reverse transcription (Invitrogen). High-throughput sequencing was performed using a HiSeq 2500 instrument. The clean reads generated by high-throughput sequencing were mapped on the grape genome (http://genomes.cribi.unipd.it/grape/) using the HISAT software (V2.0.4)^62^, and the reads mapped on the genome were assembled into transcripts using the stringTie software (V1.0.4)^63^.

### Identification of lncRNA

To identify novel grapevine lncRNA transcripts, we first filtered out all mRNA transcripts, transcripts with a length < 200 nt, and known lncRNA transcripts predicted in data from the grape genome database (http://genomes.cribi.unipd.it/grape/)^28^. Then, we predicted the protein coding ability of the remaining transcripts using the CPC^64^, txCdsPredict, and CNCI software^65^. The transcripts without protein coding ability were subsequently employed in the remainder of the study. If transcripts without protein coding ability were not found in any known domain using the pfam database^66^, we considered them lncRNA transcripts. The known lncRNAs were annotated in the grape genome database (http://genomes.cribi.unipd.it/grape/). Finally, the transcripts with FPKM < 0.5 were removed^28^.

LncRNAs that were not found near any protein-coding locus (within < 10 kb) are considered lincRNAs^5,6^. LncRNAs transcribed from introgenic regions are long intronic RNAs, which can be transcribed in any orientation relative to coding genes. LncNAT are those that overlap with protein-coding regions or ncRNAs on the opposite strand and antisense RNA^5–7^.

### Analysis of differentially expressed lncRNAs and mRNAs

DEGseq^67^ was used to identify the differentially expressed lncRNAs and mRNAs based on an MA-plot^68^. The lncRNAs significantly up-regulated (fold change > 2, P < 0.05) and down-regulated (fold change < −2, P < 0.05) under cold stress were considered the differentially expressed known lncRNAs.

### Quantitative real time PCR validation of lncRNA and protein-coding genes

Quantitative RT-PCR (qRT-PCR) was performed to analyze the expression of lncRNAs following the methods outlined by a previous study^69^. Primers used in all qRT-PCR experiments are listed in Table S8.

### Prediction of lncRNAs as miRNA targets

Target genes (lncRNAs and protein-coding genes) of grape miRNAs were identified using psRobot software set to moderate parameters (penalty score threshold = 2.5, five-prime boundary of essential sequence = 2, three-prime boundary of essential sequence = 17, maximal number of permitted gaps = 1, and position after gaps permitted = 17)^70^. Grape miRNAs were downloaded from the miRBase database (http://www.mirbase.org/).

### GO and KEGG pathway analysis

Target genes were annotated based on the GO database (http://www.geneontology.org/). Pathway analyses of target genes were performed using the KEGG database (http://www.genome.jp/kegg/kegg1.html)^71^.

### Analysis of miRNAs derived from lncRNAs

The grape precursors of the miRNAs dataset from miRbase were downloaded. The miRNAs were mapped to lncRNAs using the STAR program. The miRNAs were thought to be derived from lncRNAs if the identification between precursors of the miRNAs could be mapped to lncNRAs^28^.

## Supplementary information

Dataset 1

Dataset 2

Dataset 3

Dataset 4

Dataset 5

Dataset 6

Dataset 7

Dataset 8

Dataset 9

Dataset 10

Dataset 11
